# Contextual Choices in Online Physics Problems: Promising Insights Into Closing the Gender Gap

**DOI:** 10.3389/fpsyg.2019.00594

**Published:** 2019-03-29

**Authors:** Samuel R. Wheeler, Margaret R. Blanchard

**Affiliations:** ^1^ Department of Physics, North Carolina School of Science and Math, Durham, NC, United States; ^2^ Department of STEM Education, North Carolina State University, Raleigh, NC, United States

**Keywords:** gender, physics, online, choice, motivation

## Abstract

Throughout the world, female students are less likely than males to take advanced physics courses. This mixed-methods study uses a concurrent, nested design to study an online homework intervention designed to address choice and achievement. A choice of three different contexts (biological, sports, and traditional) were offered to students for each physics problem, intending to stimulate females’ interest and enhance achievement. Informed by aspects of Artino’s social-cognitive model of academic motivation and emotion, we investigated: Which context of physics problems do males and females select?; What explanations do students give for their choices?; Are there differences in the achievement of males and females?; and Is there a relationship between student achievement and the context selected? Fifty-two high school physics students from five US states participated. Data included pre- and post-Force Concept Inventory scores, homework context choices and achievement, and rationales for choices. Findings indicate that females were most likely to select biology contexts; males, traditional. All students made more attempts on video questions over word questions, although females did not score as well. For all questions, students generally persisted until they answered them correctly, with females taking fewer attempts on problems. Context choice was mostly driven by interest, for males, and perceptions of difficulty level for females; however, rationales were indistinguishable by gender. On their first homework question attempt, females scored significantly better than the males. Initially, males had significantly higher FCI scores; post homework intervention, females increased their mean scores significantly on the FCI, erasing the initial gender gap, with no growth nor decline in males’ scores. Females with FCI growth were equally as likely to choose biology contexts as traditional contexts; males were more likely to choose biology contexts. Findings from this study suggest that modest changes to homework problems that provide choice and make the physics problems more contextually interesting—even without changes in classroom instruction—could increase interest and motivation in students and increase achievement for both male and female students. Recommendations will be discussed.

## Introduction

In the 21st century, the demand for skilled workers in Science, Technology, Engineering, and Mathematics (STEM) is outpacing the rate at which they are produced from universities (Wieman, 2012). The National Research Council (NRC, 2013) identified physics as the ultimate foundation for all the other branches of science, with over 500,000 students a year taking an introductory physics course in the United States (US), but only 1% of college graduates completing a degree in physics. In the early grades, there is no gender gap in interest in STEM subjects for US students; yet, from the time, a young girl enters kindergarten until the time she begins her senior year of high school, chances are that she will have lost much of her interest in STEM subjects as compared with her male peers (Baram-Tsabari and Yarden, 2011). This drop-off in interest begins before students go to college (Heilbronner, 2013).

The problem with fewer female students choosing to take advanced physics courses has been documented throughout the world, including Ghana (Buabeng et al., 2012), Scotland (Reid and Skryabina, 2003), Australia (Oliver et al., 2017), England, Singapore, Spain, and Mexico (Oon and Subramaniam, 2010). In the US, approximately 36% of undergraduate STEM degrees and 19% of undergraduate physics degrees were awarded to women in 2015 (American Physical Society, 2015). Similarly, the gender gap for graduate degrees is 23% of masters’ degrees (Mulvey and Nicolson, 2014) and 21% of PhDs that are awarded go to women (American Physical Society, 2015).

The gender gap between the enrollment of male and female students in physics and the physical sciences points to three influences that place pressures on both genders to adhere to established stereotypes: cultural, attitudinal, and educational (Baram-Tsabari and Yarden, 2008). Cultural influences stem from established societal views of the “male image of science”: parental beliefs that girls are not as interested in science as are boys (particularly in the physical sciences), family responsibilities, and lack of support when in a STEM occupation. Early exposure to STEM activities and family influences have been found to contribute to long-term female student motivation to pursue a professional career in STEM fields (Talley and Martinez Ortiz, 2017).

Among the challenges that young women face in physics and engineering degree programs are microaggressions; brief, but frequent everyday interactions that send subtle but negative messages to them that they cannot be scientists or physicists (Grossman and Porche, 2014). Stereotype threat is a well-studied phenomenon that occurs when “a stereotype about an individual’s social or racial group can provide a potential explanation for the person’s poor performance” is thought to be a contributing factor to creating the gender gap in mathematics and is believed to be a contributing factor in the observed gender gap in physics (Marchand and Taasoobshirazi, 2012), p. 3051.

Attitudinal influences undermining girls’ interest in science include perceptions of the impersonal nature of physical sciences, difficulty with the material, and an image of the physical sciences as a masculine field (Baram-Tsabari and Yarden, 2008). Some assert that the gender gap in STEM is due to female student perceptions of engineers and physicists as being “nerdy” and “reclusive” people who have no time for interactions and relationships (Johnson, 2012). Females’ perceptions of educational barriers to learning and doing physics impede their full exploration and immersion in the subjects (Grossman and Porche, 2014). In addition to addressing the classroom environment and traditional pedagogy, researchers recommend making physics more personally relevant to girls (Murphy and Whitelegg, 2006; Baram-Tsabari and Yarden, 2008; Gibson et al., 2015).

Until recently, stereotypical masculine interests and characteristics were widely represented in the images and language used in textbooks with references to male names and traditionally male activities and images (McCullough, 2007). In addition to the textbooks used, validated formal assessments such as the Force Concept Inventory (FCI), one of the most widely used physics concept assessments (Hestenes et al., 1992), is largely dominated by questions from stereotypical male contexts (McCullough, 2004). These contexts lay the foundation for gender biases, which send the message to young female students that they may lack the aptitude to do well in physics or in STEM-related fields (Grossman and Porche, 2014). From the perspective of both male and female students, physics tends to be personified by masculine traits; from the teacher’s perspective, physics is perceived as having characteristics from both genders (Makarova and Herzog, 2015).

Interest and positive student motivation toward STEM subjects have been linked to the use of collaborative learning and social modeling in the classroom (Bryan et al., 2011). Sawtelle et al. (2012) found that “vicarious learning experiences,” seeing a particular task they are expected to perform modeled for them and comparing their achievement to that of others, positively influenced the development of female students’ self-efficacy in physics, a strong factor in perseverance in physics classes.

The gender gap in physics was once attributed to the assumption that the subject was too difficult for females, and programs were developed to address girls’ deficiencies (Zohar and Sela, 2003). But the gap is not due to lack of ability; female students who take physics in high school are just as likely to succeed in the course as male students (NRC, 2013). Stereotypically, male students tend to be interested in physics for the sake of physics, while female students tend to report being interested in physics for the sake of what physics can do to help humankind and other social associations (Bøe and Henriksen, 2013). Female role models in physics, such as a female physics teacher or physicist, can positively impact female students’ attitudes and interest in physics by providing someone who has a “physics identity” for female students to observe; yet, these role models are few (Hazari et al., 2010). McCullough (2007) recommends the use of specific language in physics examples and problems that involve familiar, relevant contexts for all students, such as cars, food, and school activities. Other researchers suggest tapping into the interests of female students by integrating medical and biological fields into the traditional physics curriculum (Gibson et al., 2006). In the United Kingdom (UK), a study found that female physics students wanted pedagogies that connected the relevance of physics with the greater world and with their own interests, suggesting the creation of a curriculum that relates physics to health applications and the human body (Mitrevski and Treagust, 2011).

The interdisciplinary approach to teaching physics by incorporating life science into the curriculum is on the rise, mainly as a response to the greater demand for students to more fully understand the relevance of physics in relation to biology and chemistry (Crouch and Heller, 2014). Crouch and Heller designed a course for the growing number of life science majors who need physics, to deliver a “coherent view of physics as a discipline” (p. 379). Others have recommended the integration of biology and physics in university courses to begin recognizing the similarities of the two disciplines instead of the differences (Hoskinson et al., 2014). One study found that by incorporating topics and phenomena that students do not encounter in everyday life into the physics curricula, students become more interested in the physics concepts (Badri et al., 2016). The Badri et al. (2016) study found that females were more interested in phenomena that could not be easily explained by high school physics, while males were more interested in traditional phenomena such as mechanical equipment and lasers.

Giving students choice in their assignments or classroom is thought to be a key factor in supporting and fostering intrinsic motivation (Patall et al., 2008). One study investigated giving students three choices for a task and found that participants who were already interested in a concept or topic showed more motivation and better performance on the task when given the opportunity to choose, which did not happen for disinterested students (Patall, 2013). Patall et al. (2010) concluded that performance and engagement stemmed from intrinsic motivation to complete the task. Thus, giving students choice is a key factor in supporting and fostering intrinsic motivation. Others have also found that choice can be a motivating factor when the choice is meaningful, relevant, and enhances the competence of the student (Evans and Boucher, 2015).

Teachers with rich content knowledge and enthusiasm toward teaching can result in positive gains in student motivation in physics (Keller et al., 2017). One recent study showed that female students’ motivation to study and do well in physics is linked to several factors, including having a combination of teachers, supportive and knowledgeable teachers, engaging pedagogy, the school’s science culture, and social interactions with family and peers (Oliver et al., 2017). In another study, students’ motivation in physics was positively related to the task-value they saw in the physics they were doing and interest in the science being studied (Wang et al., 2017). Similar results were found in a Croatian study, which suggested that a key motivational factor for female students was perceptions of its utility value for students (Jugović, 2017).

Another important factor in learning physics is how students comprehend a range of multimedia representations, such as visual pictures (e.g., a bar graph or photo), visual texts (written information), and sound (Schnotz, 2014). Learning occurs when an individual understands what is presented; that is, “when the individual uses external representations in order to construct internal (mental) representations of the learning content in working memory and if he or she stores those in long-term memory” (p. 75). Mayer’s generative theory of textbook design (Mayer and Sims, 1994; Mayer et al., 1995) focuses on the relationship between illustrations in textbooks and the corresponding text. Illustrations, photos, drawings, and animations are examples of visualizations, a type of multimedia representation involving spatial relations that communicate information (Scheiter et al., 2009). Mayer et al. (1995) found that students received higher scores when illustrations were accompanied by text in close proximity. The use of pictures and illustrations most enhances student learning when the image and the information from the text are integrated, compared to text only (and the complexity of the diagrams influences the outcome) (Mason et al., 2013; Jian and Wu, 2015). The learning is enhanced when the words and pictures are semantically related, if they are presented close together in space or time, and when the picture appears before the text (Schnotz, 2014). Using their spatial ability helps students to consolidate and clarify ideas, remember ideas, and helps with problem-solving (Baker and Pilburn, 1997).

The use of videos as pedagogical tools was the next logical step from diagrams and photographs and was originally seen as a way to introduce concepts to students that would motivate them to explore the concept further, to understand more, and to examine “what if” questions—therefore allowing them time to bridge the gap between the abstract and the concrete (Zollman and Fuller, 1994). Videos and video analysis technology as pedagogical tools were introduced over 25 years ago to more effectively teach kinematics and help students better understand the physics of motion (Beichner et al., 1989), and has been found to increase student excitement and engagement with the material being presented (Lee and Sharma, 2008).

The development of additional video analysis software (e.g., Vernier’s Logger Pro^©^, Pasco’s^©^ commercial versions) and other technologies developed specifically for the physics classroom provide students with the ability to collect real-time data, which can motivate them to want to learn the underlying physics concepts and also provide a way for them to more easily clarify and correct their misconceptions about motion (Beichner and Abbott, 1999). Struck and Yerrick (2010) found that video analysis as the sole lab technology more effectively promoted student comprehension. However, interactivity with a computer and controls on an animation or dynamic displays as well as inaccurate prior knowledge can reduce comprehension (Hegarty, 2014).

## Theoretical Framework

**Figure 1:**
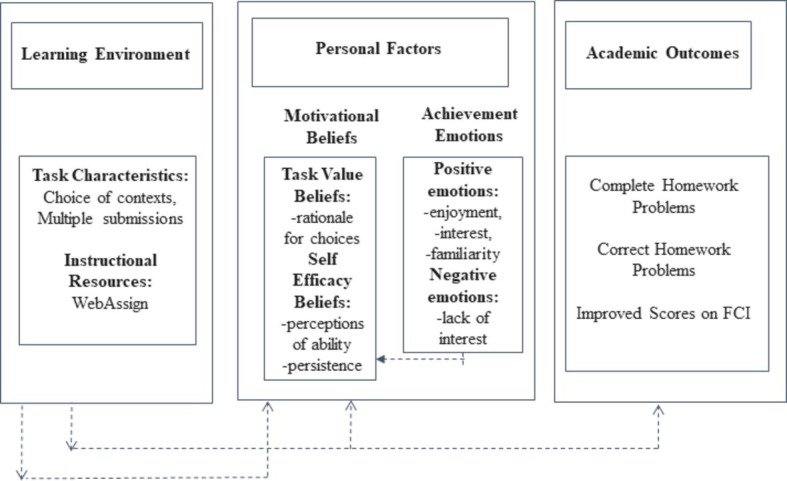
Predicted social-cognitive model of achievement motivation and emotion (adapted from Artino, 2010).

This study was guided by Artino’s (2010) social-cognitive model of achievement motivation (Figure 1). Based on his work with at-risk students in an online setting, Artino found that students who were more satisfied with their experiences and more confident in their abilities were more likely to prefer taking online courses in the future. In his model, the learning environment and motivational beliefs contribute to (dis) satisfaction and academic outcomes. Students’ motivational beliefs are directly linked to student self-efficacy or one’s beliefs about the task’s interest and significance, which will determine his or her motivation for completing that task (Eccles and Wigfield, 2002). Phillips (2015) used Artino’s model to investigate motivational factors of at risk students who worked on self-paced, online modules during summer school to remediate a failed science course. She found that most of the students were satisfied with the opportunity to set their own pace and take control of their learning, and all of the students achieved passing grades for their courses (Phillips, 2015). Achievement emotions describe the feelings (e.g., boredom, enjoyment) that are the direct result of achievement outcomes experienced during learning, which influence self-efficacy beliefs and task value beliefs (Pekrun, 2006). The model predicts that students’ motivational beliefs and achievement emotions are linked to their academic outcomes and are influenced by the learning environment.

When translating the context of this study into Artino’s (2010) model (see Figure 1), the *Learning Environment* includes the instructional resource WebAssign, and the task characteristics of the homework assignment are students’ choice of contexts and the ability to re-take the items, use multiple contexts, and take the needed time for both word problems and video problems. Under *Personal Factors*: *Motivational Beliefs*, the students provided a rationale for the homework contexts that they chose, some of which related to perceptions of their ability, and students were allowed to persist up to five attempts on the problem. Under *Personal Factors: Achievement Emotions*, students could report such aspects as enjoyment, interest, familiarity, or lack of interest to explain their choices. The *Academic Outcomes* considered in this manuscript were completion of and success on homework problems and (improved) scores on the Force Concept Inventory (FCI).

## Research Questions

The main objective for this study, guided by our theoretical framework, was to examine choices students made during a research-designed, online physics homework intervention focused on Newton’s Laws and their applications. Therefore, the research questions guiding this study were:

(1) When given problem choices designed around gender stereotypes, which types of physics problems do males and females select? (2) What explanations do students give for the problem contexts they select? (3) Are there differences in the achievement of males and females?, and (4) Is there a relationship between student achievement and the context of physics problems they select?

## Materials and Methods

### Research Design

This mixed-methods study was designed to help us understand more about the role of gender on choice and achievement for high school physics students. Students were given choices of contexts that were designed by the first author, based on research for how to improve females’ interest in physics. This study used a concurrent, nested (embedded) design (Creswell et al., 2003); the main data collection was quantitative data, but nested qualitative data was gathered to understand students’ rationales for their choices.

### Recruitment and Participants

Fifty-two students, 21 females (40.4%) and 31 males (59.6%) representing eight different schools from five states in the United States (US), took part in this study (53.8% white, 32.7% Asian, 9.6% Hispanic, 1.9% Native American, 1.9% other). All of the students were currently enrolled in Honors Physics or AP Physics at their schools. All the students had completed a unit on Newton’s Laws and their applications prior to this study. The Institutional Review Board (IRB) at the university approved the proposed study.

### Homework Problems

The sets of physics problems used in this study were delivered to the students through the online homework delivery system WebAssign.^1^ The WebAssign problem set instrument consisted of a total of 21 problem sets made up of 16 word problems, and 5 video analysis problems, all of which were developed by the first author (for additional detail see Wheeler, 2017). Each set of problems (word and video) contained three questions of equivalent difficulty and covered the same physics concept but using different contexts (i.e., sports, biological, or traditional). The homework questions were validated and vetted by current physics teachers to ensure equivalent difficulty level, content, and consistency of the intended contexts. WebAssign was provided freely to all study participants.

**Figure 2:**
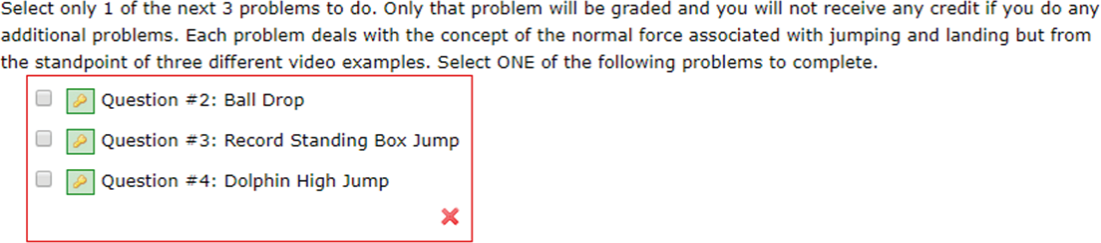
Sample view of context choices in WebAssign.

Each problem set consisted of the same fundamental physics problem but from the perspective of three different format contexts: traditional (e.g., ramp, ball, pendulum), sports (e.g., baseball, basketball, extreme sports), or biological (e.g., frog, cat, leopard). WebAssign was programmed so that students were first presented with three different scenarios to choose from, without being able to see the actual question. For example, the student was shown that the question was about the concept of net forces and asked which context they wish to choose to investigate the concept: mass and spring, the high jump, or the baby bird. Figure 2 shows an actual view in WebAssign that the student would see. WebAssign collected data on the students’ context choices, the order of those choices, and whether the responses were correct. Students received immediate feedback from WebAssign on each question they answered as to whether their answers were correct or not (either a green check if the answer was correct, or a red “x” if the answer was incorrect).

### Student Rationale

After completing each question, students were asked to give the reason (rationale) for why they chose the particular question context by writing a short answer response in WebAssign. Codes were developed by the first author by reading through the (blinded) student responses and creating categories that corresponded to their choice explanations. Once the initial categories were developed, they were collapsed into similar categories (5). An independent physics education researcher took students’ responses and coded them using the researcher-established (first author) five codes: (1) Interest, (2) Familiarity of the problem, (3) Random Choice, (4) No preference, and (5) Easy/straightforward. Differences between the results were discussed and resolved after careful evaluation of the coding scheme in which two of the codes (3—random choice and 4—no preference) were collapsed into one. After the refinements, the final inter-rater reliability was found using Cohen’s kappa to be 0.875 (87.5%) agreement.

### Force Concept Inventory

Conceptual understanding of Newtonian concepts was measured, before and after the delivery of the problem sets, by using a 29-item Force Concept Inventory (FCI). The FCI was developed in the early 1990s by physics educators who saw the need to more fully understand student misconceptions in order to design more effective introductory physics courses (Hestenes et al., 1992). The Force Concept Inventory was designed specifically around what the authors called “common sense alternatives” to actual Newtonian physics since, as Hestenes et al. (1992) found, many students coming into introductory physics courses fail to grasp Newtonian concepts but instead rely on their own misconceptions and beliefs that do not match scientific explanations. The FCI has been validated and found to be a reliable tool for identifying how much students understand about the physics concepts of Newton’s Laws and forces (Hestenes et al., 1992). Although there have been concerns about the gender bias of the items as being dominated by questions from stereotypical male contexts (McCullough, 2004; Grossman and Porche, 2014), as this was the most widely used instrument available that was linked to content focus of the unit, it was selected to measure students’ pre and post content knowledge.

## Findings

### Physics Problems Selected by Males and Females

The first research question investigated the types of physics problems males and females selected. Overall, the females were more likely to choose the biology context (37.6%) over the traditional or sports context (see Table 1). In the “Female 1st Choice Combo” category, females who chose to complete combinations (more than one context selected, per question) were more likely to choose to do the biology context (39.7%) over the other contexts. Female students who did not choose to complete a second context were more likely to choose the traditional context (37.6%) over the biology (34.9%) or sports context (25.9%). Females who chose to complete multiple contexts tended to select more traditional contexts throughout the project and fewer sports contexts, while their choices of the biology context remained relatively constant. Females who chose only one context tended to select more biology contexts and fewer traditional contexts across the project, while the number of sports contexts remained constant. The breakdown of each category, by percentage, is recorded in Table 1. Twelve (57%) female students chose more than one context to complete. The highest rate of context pair choices for female students was the traditional/sports context pair choices. Females’ second highest combination was completing every context (traditional, sports, and biology) in the problem.

**Table 1:** Student choices of question context by gender.

Group	Number of students	Traditional contexts chosen		Biology contexts chosen		Sports contexts chosen	
		Total	%	Total	%	Total	%
Female (overall)	21	154	34.9	166	**37.6**	116	26.3
Male (overall)	31	253	**38.9**	216	33.2	176	27.0
Female 1st choice combo	12	83	32.9	100	**39.7**	67	26.6
Male 1st choice combo	21	168	**38.1**	147	33.3	121	27.4
Female No Combo	9	71	**37.6**	66	34.9	49	25.9
Male No Combo	10	85	**40.5**	69	32.9	55	26.2

Each row may not add up to 100% because some of the options were not selected, although this only represents fewer than 2% of the choices.

Note: Highest values for each group are bolded.

Overall, the males were more likely to choose the traditional context (38.9% of the time) as compared with biology context (33.2% of the time) and sports (27% of the time). Males chose the traditional contexts 38.1% of the time (as part of a combo), and 40.5% of the time (traditional without a combo), with approximately the same rate of choice for biology (with combo 33.3%; without combo 32.9%) and sports (with combo 27.4%; without combo 26.2%). Males who selected multiple contexts tended to select more biology contexts and fewer sports questions, while the number of traditional contexts selected remained relatively low. Males who chose only one context tended to select more traditional questions and tended to select slightly fewer sports and biology contexts across the duration of the project. Overall, males were most likely to choose traditional contexts (38.9%) (see Table 1).

Males were more likely to choose multiple contexts within one problem set than females (males, 72; females, 37); however, there were more male participants than female participants. When looking at the percentage of choices by gender, 57% of females chose multiple contexts and 68% of males did. The highest rate of context pair choices for female students was the traditional/sports context pair choices. Male students’ highest rate of choice for context pairs was for biology/sports. Males’ second highest combination was completing every context in the problem. Less than 2% of the questions were not answered by each group and did not significantly affect the total.

### Students’ Explanations for the Question Contexts That They Selected

The second research question addressed students’ rationale for the choices that they made. Students’ written explanations for problem context choice differed by gender. Males were more likely to select a question context based on interest, such as “I like leopards” (males 45% of the time vs. 34.3% females; *p* < 0.001; Cohen’s *d* = 1.1). Females were more likely to choose a problem because they thought it looked easier (32.6% of the time; males 25%; *p* = 0.026; Cohen’s *d* = 0.306), with no significant differences between males and females on problem selection due to it being familiar, such as the “type of mechanics I am most familiar with” (17.6% males; 12.3% females) or just a “random” choice (15% males; 15.6% females). As you can see in Table 2, the written explanations by male and female students were quite similar.

**Table 2:** Sample male and female responses for choosing video problems (VP) and word problems (WP).

Gender	*Student response*	% of gender who chose question
5V(Tr) Video problem deals with circular forces (ball in loop, running man in loop, car in loop)		
M	The ball in a loop seemed kind of borinVg, and the car in a loop isn’t that impressive, but a human running in a perfect loop is pretty cool. I enjoyed seeing the 5 second video and was motivated to actually complete the problem because of it.	57.1
F	Because it’s more impressive than the others, and cool to watch and think about. ITS GOT A HUMAN and its cool! I think I did it the correct way and I have looked through my notes to find it.	42.9
10V (Sp) Video problem about friction/resistive forces (arrow in gel, plane landing, frog jumping)		
M	I choose this because I shoot bow and arrow sometimes and I thought it was cool. The context was interesting, as I have not seen an object slow in its velocity due to a solid.	62.5
F	A jumping frog is easy to picture. I feel more confident calculating parabolas.	37.5
3(Bio) Word problem deals with tension in a taut line (spider & thread; mountain climber & rope; elevator & cable)		
M	The problem, as I read it, was extremely straightforward and I knew what I needed to do to solve it immediately. I thought that incorporating “spider” and “fly” made it interesting	75
F	This problem was straightforward and easy to understand. The context was interesting. Animals make the problem more relatable and “friendly of sorts”.	25

### Differences Between Males and Females on Homework Submissions and Success

The third research question investigated differences in achievement for males and females. First, the homework questions that were completed on WebAssign were examined. For all of the homework questions, students generally persisted until they answered them correctly (up to five attempts allowed), and a majority of the students took advantage of the opportunity to choose different (and therefore, multiple) question contexts (55% of females; 68% of males). Students were more likely to make attempts on video problems (V) versus the word problems (W); on video problems, males averaged 2.52 attempts on traditional (VTr) contexts (vs. 2.3 WTr) and 3.38 attempts on biology (VBio) contexts (vs. 2.52 WBio) (see Table 3). Females averaged from 2.78 attempts on video sports contexts (vs. 2.23 WSp) to 3.02 attempts on traditional contexts (vs. 2.24 WTr). Although females were most likely to choose biology contexts, overall (37.6%), and most likely to choose biology as a first choice (39.7%) (Table 1), they had the fewest number of *attempts* to answer the biology context questions (1.88; Table 5), suggesting that they were more likely to answer these questions correctly with fewer attempts than with other contexts (i.e., 2.24 attempts Tr, 2.23 attempts Sp).

**Table 3:** Average percentage of responses correct and # submissions by category of video questions.

	Traditional contexts video		Biology contexts video		Sports contexts video	
Gender	% correct	# submissions	% correct	# submissions	% correct	# submissions
Females total	66.0	3.02	**69.8**	2.80	45.7	2.78
Males total	71.5	2.52	**73.0**	3.38	53.5	2.99
Females who chose a combination						
Video Qs	**72.6**	2.58	71.1	2.47	55.2	2.17
Word Qs	81.8	2.09	**86.8**	1.78	70.4	1.98
Males who chose a combination						
Video Qs	**71.0**	2.58	67.3	3.34	58.0	2.56
Word Qs	**81.0**	2.23	79.5	2.21	1.90	2.23
Females who did not choose a combination						
Video Qs	57.1	3.57	**73.5**	3.41	29.4	3.82
Word Qs	64.8	3.04	**92.6**	2.41	58.1	3.35
Males who did not choose a combination						
Video Qs	78.0	2.16	**88.2**	3.29	50.0	3.33
Word Qs	**92.4**	1.59	91.3	1.83	85.5	1.84

Note: Highest values for each group are bolded.

As shown in Table 4, for both males and females, students scored highest (correct response on homework question) on the biology contexts than any other context. This trend follows with the traditional context, which is the second highest score correct for every gender and group, and then the sports contexts (which had the lowest score correct for each gender and group). The number of submissions did not follow such a simple pattern, but the general trend was that questions that had more submissions suggest that the student stayed with the problem longer, while fewer submissions seems to suggest the student either got the problem correct more quickly or gave up on the problem earlier. For both females who did multiple contexts and those who did not, the number of submissions for the biology context is smaller compared with the other contexts. The high score on the biology contexts and the low number of submissions indicate that females had an easier time with the biology context questions than the other contexts.

**Table 4:** Average score of all responses and submissions by category.

	Traditional contexts		Biology contexts		Sports contexts	
Gender	% correct	# submissions	% correct	# submissions	% correct	# submissions
Females who chose a combination	73.7	2.24	**83.1**	1.88	69.6	2.23
Males who chose a combination	74.9	2.29	**76.3**	2.52	72.0	2.35
Females who did not choose a combination	59.9	3.17	**86.9**	2.65	52.7	3.63
Males who did not choose a combination	85.1	1.80	**91.3**	2.10	77.4	2.40

Note: Highest values for each group are bolded.

### Male and Female Achievement on the Force Concept Inventory

In order to investigate any differences between achievement on the Force Concept Inventory (FCI), scores of females pre (*M* = 15.95) and post (*M* = 18.2) were compared and found to be significantly different in their performance (*p* = 0.004; Cohen’s *d* = 0.359). There were no significant differences in the pre (*M* = 20.9) and post (*M* = 20.5) scores for males (*p* = 0.298; Cohen’s *d* = 0.055; see Table 5).

**Table 5:** Pre/Post FCI comparisons within and between gender.

Group	Pre-FCI Avg	Post-FCI Avg	Δ Avg	t	df	*p* (one-tailed)
Pre/Post FCI comparisons within gender						
Female	15.95	18.2	2.25	−2.986	20	0.004**
Male	20.84	20.48	−0.36	0.535	30	0.298
Pre FCI comparisons female to male						
Female	15.95					
Male	20.84			−2.718	42	0.005**
Post FCI comparisons female to male						
Female		18.2				
Male		20.5		−1.266	47	0.106

** p ≤ 0.01.

Next, the FCI scores for the females and males were analyzed using a two-sample *t*-test assuming unequal variances and summarized (Table 5). Males’ pre-FCI scores (*M* = 20.6) were significantly higher than those of the females (*M* = 16.9) in the pre-FCI, but this gap was closed by the post-FCI (females 18.4; males 20.3; *p* = 0.155; Cohen’s *d* = 0.354).

In order to further examine the differences between the FCI scores of males and females, these groups were subdivided into a “high” group (FCI scores of 21–30) and a “low” group (FCI scores 0–20) for males and females. The mean pre-FCI scores of the females (*M* = 13.4) and males (*M* = 14.4) in the “low” FCI group were not different (*p* = 0.292; Cohen’s *d* = 0.21); nor were the mean scores of the low post-FCI group (Females: *M* = 16.5; Males: *M* = 14.2; *p* = 0.168; Cohen’s *d* = 0.379). The mean pre-FCI scores of the females (*M* = 24) and the males (*M* = 24.9) in the “high” group were not different (*p* = 0.257; Cohen’s *d* = 0.359); nor were the post-FCI scores of the high group (Females: *M* = 23.7; Males: *M* = 25.3; *p* = 0.065; Cohen’s *d* = 0.656).

Next, the FCI scores of males and females whose FCI scores increased (growth groups) were analyzed in two ways. First, both the pre and post scores of growth males and growth females were compared. As can be seen in Table 6, males and females in this group were significantly different from each other, both pre- (*p* = 0.0115; Cohen’s *d* = 0.985) and post-FI (*p* = 0.0114; Cohen’s *d* = 0.977). Next, the pre- and post-FCI scores of females were compared, as were the pre- and post-FCI scores of males (see Table 7). There were significant increases in the growth for females (76% of females improved), from pre- to post-FCI (Female Pre: *M* = 14.6; Post: *M* = 18.1; *p* = 0.0466; Cohen’s *d* = 0.613). When the pre- to post-FCI scores for the male growth group (35% of males improved) was examined, there were not significant differences (Male Pre: *M* = 20.9; Post: *M* = 23.7; *p* = 0.163; Cohen’s *d* = 0.430).

**Table 6:** Comparisons of FCI growth groups only FCI scores.

Group	Female	Male	T	df	*p* (one-tailed)
Pre FCI	14.6	20.9	−2.47	19	0.0115^*^
Post FCI	18.1	23.7	−2.47	20	0.0114^*^

* p ≤ 0.05.

**Table 7:** Comparisons of pre and post FCI scores for growth groups only.

Group	Pre FCI	Post FCI	t	df	*p* (one-tailed)
Female	14.6	18.1	−1.73	30	0.0466^*^
Male	20.9	23.7	−1.01	20	0.163

* p ≤ 0.05.

### Student Achievement and the Context of the Selected Physics Problems

For the final research question, the students’ success on homework problems were analyzed based on the context of the homework problems they selected. Students could select the same context more than once or select a different context from the choices of biology, sports, or traditional. The data in Table 8 display the first, second, and third context choices of males and females, overall, the average number of correct responses, and the mean number of submissions. The means were compared using two-sample *t*-tests, assuming unequal variances. Females who chose multiple contexts per question had significantly more correct responses (*p* = 0.013; Cohen’s *d* = 0.441) on their first choice and achieved that with significantly fewer submissions (*p* ≤ 0.001; Cohen’s *d* = 0.885) than male students who also chose to complete multiple contexts.

**Table 8:** Average score of responses and submissions by gender.

	First choice		Second choice		Last choice (for ‘all’ category)	
Gender	% correct	# submissions	% correct	# submissions	% correct	# submissions
Female	85.1^*^	2.11	40.5	2.30	33.3	2.89
Male	69.5	3.22^***^	47.2	2.49	25.0	2.55

* p ≤ 0.05;

*** p ≤ 0.001.

Next, females and males were divided by those who chose a combination of different contexts, and those who did not. As you can see in Table 9, all students were most likely to answer the biology context problem correctly, followed by the traditional context, and then the sports context. The mean number of submissions (“tries”) on each problem indicated how many times a student attempted the problem. Female students had a lower number of submissions for the biology context. The high score on the biology contexts and the low number of submissions suggest that female students had an easier time with the biology context questions than the other contexts. To determine whether these differences were significant, the mean percentages of their correct questions (in Table 9) were compared using two-sample *t*-tests, assuming unequal variances.

**Table 9:** Average score of responses and submissions by category.

	Traditional contexts		Biology contexts		Sports contexts	
Gender	% correct	# submissions	% correct	# submissions	% correct	# submissions
Females who chose a combination	73.7	2.24	**83.1**	1.88	69.6	2.23
Males who chose a combination	74.9	2.292	**76.3**	2.52	72.0	2.35
Females who did not choose a combination	59.9	3.17	**86.9**	2.65	52.7	3.63
Males who did not choose a combination	85.1	1.80	**91.3**	2.10	77.4	2.40

Note: Highest values for each group are bolded.

Overall, students were most likely to choose a biology context, most likely to get that choice correct, and generally it took the fewest number of submissions to get those homework problems correct. Virtually all of these differences were found to be significantly different from one another. Females scored significantly better (85.1% correct) on their first choice than did males *p* ≤ 0.05, (Cohen’s *d* = 0.441) regardless of context. Females also had significantly fewer submissions (2.11) on their first choice than did males (3.22) *p* ≤ 0.001, (Cohen’s *d* = 0.885) regardless of context.

Results on the video and word problems were also investigated to see if there were differences in choice and success on these problems. Overall, males and females were most likely to get the biology context questions correct, although they were less successful on all of the video problems than they were on the word problems, with an average success rate for females 69.6% (2.8 attempts) and males 73.0% (3.38 attempts). For the video questions, the patterns for males and females were similar, although the males tended to make more attempts on problems, and the variation between nearly every choice group (combination/no combo), traditional, biology, sports, and male/female was significant (Table 10).

**Table 10:** Average percentage of responses correct and # submissions by category of video questions.

	Traditional contexts video		Biology contexts video		Sports contexts video	
Gender	% correct	# submissions	% correct	# submissions	% correct	# submissions
Females total	66.0	3.02	**69.8**	2.80	45.7	2.78
Males total	69.6	2.52	**73.0**	3.38	53.5	2.99
Females who chose a combination	**72.6**	2.58	71.1	2.47	55.2	2.17
Males who chose a combination	**71.0**	2.58	67.3	3.34	58.0	2.56
Females who did not choose a combination	57.1	3.57	**73.5**	3.41	29.4	3.82
Males who did not choose a combination	78.0	2.16	**88.2**	3.29	50.0	3.33

Note: Highest values for each group are bolded.

Student achievement on FCI scores were investigated in connection to the context choices made by females and males. Males who had FCI growth chose significantly more biology contexts than did males without growth (*p* = 0.040). Males who had no growth on the FCI chose significantly more traditional contexts than biology (*p* = 0.002). Females who showed FCI growth chose as many traditional contexts as biology and had more growth than those females who chose sports contexts. There were no differences on FCI scores based on the number of context choices females selected.

## Limitations

Our findings need to be viewed in light of several limitations. First, we are unable to rule out all potential alternative explanations for the observed differences due to the nature of the research design. Our choice of outcomes and how we decided to measure them provides us with only a limited picture of what the students experienced during the WebAssign physics unit. Our findings, as a result, may have differed if we chose to target different learning outcomes. Second, the number of participants in this study was small and the nature of their experience during the intervention was unique and did not consider socioeconomic status nor students in lower-level courses. Additionally, the number of males and females also was unequal—typical of physics classrooms and part of the rationale for this study. The generalizability of our findings, therefore, might be limited to this single case. Third, we only analyzed one physics homework unit. Students had already learned about Newton’s Laws, and they were in eight different classrooms across the US, which were not directly observed. Differences in time were unable to be clearly understood from the data in WebAssign, and we do not know whether students used additional help, although the problems were developed for this study and not available online. We therefore cannot make any claims about their direct experiences or other learning or interest in physics, nor whether these findings would be the case for a different population of students. With these limitations in mind, we will now discuss the findings of this study in light of the available literature.

## Discussion

### Differences Between Females and Males in Context Selections

The context choices made by male and female students were analyzed to answer the first research question, “When given assignment choices designed around gender stereotypes, which types of physics problems do males and females select?” Females, overall, chose biology contexts more often than the other two contexts, traditional and sports. Male students, overall, were more likely to choose the traditional context over the other two contexts. Although no other studies have conducted a similar online intervention, nor in a physics problem set or providing the choice of a sports context, the findings are resonant with those of Baram-Tsabari and Yarden (2008). In their work, Baram-Tsabari and Yarden studied the interest of females and males, of various ages, toward biology and physics and similarly found that females were significantly less interested in traditional physics than the males, but females were significantly more interested in biology than male students. Baram-Tsabari and Yarden’s (2008) study asked children, adolescents, and adults to create sets of self-generated questions that were classified according to interest in biology or physics.

An unexpected but interesting affordance of the WebAssign technology was that students could choose one or more contexts on additional attempts of the same problem. Although students were told at the beginning of the assignment that they would not receive any extra credit for completing extra assignments, most students chose to answer multiple contexts (and thus, complete extra problems). The software allowed the researcher to track the pattern of choices of the students. Those males and females who chose to do multiple contexts within a single question were placed into subcategories for analyses; females with combinations (“combos”) and males with combos, regardless of how many multiple contexts they chose to do. Females reported they chose a certain context for two main reasons: because they were interested in the context (34% of the time) or because they thought the context presented looked easy (33% of the time). As a reminder, the students were asked to select the question context prior to seeing the actual physics question. That is, a student might have chosen a “cuddly kitten” (interest) or “the ball drop” (easy) prior to actually seeing and then attempting the problem, which they could view after this selection. However, students then reported their rationale for the choice after completing each problem.

The trend of context choices over time for females with combos shows that they had a constant rate of selecting biology contexts throughout the project, a slightly increasing rate of selecting traditional contexts, and that they chose sports contexts at a decreasing rate from the beginning of the project to the end. Females who chose to complete multiple contexts were more likely to choose to complete traditional-sports combinations or answer all contexts, followed by traditional-biology, and biology-sports combinations, which were selected at equal rates. Females who chose only one context tended to choose slightly more biology questions, fewer sports contexts, and chose traditional contexts at a relatively constant rate.

These context choices were explained by students with written comments such as, “It looked like something we did in class,” “I like kittens,” or “It looked easy.” Female students were equally likely to write that they chose questions because they were interested in the context, or they thought the question was easy. Therefore, the given context of the question led to students’ perceptions of these factors. Similar to this study, in which the researcher designed the choice options (biological, traditional, and sports), the Patall et al. (2010) study used teacher-determined written assignments given to high school students in chemistry, biology, and history classes, who chose which assignment to complete. Patall et al. (2010) found when students had a choice of homework, they had higher intrinsic motivation to complete the assignment and felt more competent doing the assignment. Given their continued choices, it seems likely that providing choices was perceived by students as motivating.

It seems that the students’ perceptions about the choices that they made were consistent through the end of the problem set, even though several students correctly noted that the different contexts of each question were essentially the same problem, just presented in a different circumstance.

### Video Questions

Compared with the written questions, both male and female students were more likely to choose to complete multiple contexts with video questions than they were word problems. The main reason why students chose to complete a video question was because of interest in the context or the “cool” factor. Students described the reasons they selected the video problems with responses such as “I enjoyed seeing the 5 second video and was motivated to actually complete the problem,” “the graph made it easy to utilize the values in the problem,” and “I found it most interesting.”

Students were interested in the video analysis questions enough to try multiple contexts and they were interested in the written questions #3 (which dealt with tension in a taut line and included the choices of a spider and thread, mountain climber and rope, or elevator and cable), and #13 (which dealt with the normal force experienced when something hits the ground and included the choices of a rocket lift off, a cuddly kitten jump, or a standing high jump), seemingly because of the examples used in the contexts. Based on the response rate and student comments, the video format was more engaging to all students than the word format.

### Female and Male Achievement Differences

To answer the question, “Are there differences in achievement of males and females and is this related to the types of physics questions they select?” achievement in this study was measured through FCI scores and WebAssign scores. First, FCI scores will be discussed. Females made significant gains on the post-FCI as compared with their pre-FCI, and they closed the gender gap with males by the end of the problem set. Female students tended to choose biology contexts more than male students and most female students chose to complete a second context. Females did better on the biology contexts than any other context in either word or video format. Overall, students were limited to the context choice, the time they spent on the problem, and the number of submissions per problem as the only variables that they could manipulate in this study. McCullough (2004) argues that the FCI is dominated by questions that align with stereotypical male contexts. In the findings of this study, females not only significantly improved from their pre- to their post-FCI score, but their post score was statistically equal to the males’ post score. Given these findings and the research of McCullough (2004), it is possible that the intervention may have been even more successful at improving female students’ conceptual understanding than first thought.

In this study, all of the choice options afforded to the students were equally difficult. This has not necessarily been the case with other studies in the literature, which have also found that giving limited choice in the type of assignments or homework students can result in greater gains on assessments. Fulton and Schweitzer (2011) found that giving students a choice in the type of final class assignment (one defined as easier, the other more challenging) in a computer science class resulted in lower performing students choosing an easier assignment option and therefore learning less than their peers. However, similar to this study, in a meta-study literature analysis on the effects of choice in a variety of settings, Katz and Assor (2007) found that choice motivates students when the choices are limited and aligned with student interests and goals.

In contrast to the female students, males did not show the same kinds of gains and had no significant differences between their pre- and post-FCI scores, even though males had the highest scores on the traditional context questions of the problem set. The intervention appeared to have had a positive effect on female students’ performance on the FCI but not the males’ performance. Why might this have happened? Even more so than the females, males were more likely to choose a question to work on because it looked interesting to them (45%). One difference from the females was that males who chose to do multiple contexts tended to choose traditional contexts at a constant rate throughout the project, and they chose fewer sports contexts and slightly more biology contexts throughout the project. Indeed, males who chose to complete only one context tended to choose more traditional contexts from the beginning to the end of the homework set. Males selected slightly fewer sports contexts across the project but chose biology contexts at a constant rate. The most common combination of contexts were biology-sports, then all three contexts chosen together, followed by traditional-biology, and traditional-sports.

The effect of the choice of context on female students’ FCI growth can be seen in the feedback loop of Artino’s (2010) framework, in which positive emotions such as interest or enjoyment resulted in positive academic outcomes such as achievement and conceptual understanding. For males, the feedback was that they were more likely to get the traditional context problems correct compared with the other contexts, which may have resulted in more confidence in doing the problems and more satisfaction which reinforced their choice of traditional contexts. The homework questions were open response, not multiple choice, so the students needed to complete the problems to get credit instead of just checking a box. It is expected that the more time a student spends on an assignment or the more problems they complete, the more their understanding will grow from exposure to the content.

There was not a significant difference between the pre- and post-FCI scores of males who chose to do combos versus males who chose only one context; however, males who chose one context had a higher absolute score on both measures. Indeed, traditional physics courses have privileged male students (Baram-Tsabari and Yarden, 2008), and the traditional choice also was the one more likely to correspond to an example given in the classes of the teachers who helped with this study (as explained in the Methods section).

Students who chose combos, regardless of gender, tended to do better on their first-choice question context. Interest in the context seemed to be more of a deciding factor for initial choice rather than the perceived difficulty level (e.g., choosing a problem because it seemed “easier”). Even though the questions were essentially the same, except for the context, students reported that one context seemed easier than another, suggesting that interest impacted perceptions of difficulty and perhaps making the problem seem more relevant, and therefore more manageable.

### Homework Question Format: Word and Video

The homework problems had an additional “wrinkle.” They were either word problems or video problems. The video questions were novel from what the students had seen in their classes (based on communication with the teachers), but they also required students to gather data from the video in order to try to solve the problem. Therefore, there were some additional and unfamiliar steps involved in trying to solve those problems. Significant differences were found between the performance of males on the video questions and the word problems. Males, regardless of multiple context choice or not, performed better and used fewer submissions on the word problems compared to the video problems for each context (biological, traditional, or sports). Although males indicated that they enjoyed the video questions more than the word questions, this did not lead to better performance on those problems. Males who selected multiple contexts scored significantly higher on the biology video questions than did males who chose only one context.

Results from the female students on the video questions were not as clear. Overall, females who completed combos tended to have higher mean FCI scores and higher scores on traditional and sports questions than female students who did not choose a combo, although these differences were not significant. Females who did not choose a combo tended to have higher scores on the biology contexts compared with females who chose to complete multiple contexts.

## Conclusion

This study adapted research regarding student interests and choice into an online physics assignment designed to investigate their possible role in the attitudes, understanding, and achievement of male and female students. A number of conclusions can be drawn from the findings of this study. From the findings, we see that when given problem choices designed around gender stereotypes, females were more likely to choose questions related to biology contexts, while males were more likely to select traditional physics contexts. Females were more likely to indicate that they selected a problem because they thought it looked easy, while males indicated they selected a problem because they were interested in it. When offered choice, many males and females chose to complete multiple contexts within a problem. Females and males who chose multiple contexts had higher scores on the biology problems than the other contexts. There were significant improvements in female post-FCI scores as compared with pre-FCI scores but no difference between pre- and post-FCI scores were found for the males.

We investigated whether there was a relationship between student achievement and the context of physics problems they selected. First, students made many choices of different contexts, suggesting that they were motivated by the ability to choose. The students who were more likely to take advantage of these combinations of problems were those who selected biology contexts over traditional contexts. Second, males and females were both motivated by interest, but this reason was more likely to be stated by males. Third, students persisted in problems, as shown by the number of submissions made on each problem, that were more challenging or with which they were less successful when they were motivated by interest or novelty, as they were in the video questions. Fourth, the intervention led more females than males to improve their scores and erased the initial gender gap seen on the FCI scores, pre-intervention. Fifth, even a short-term, online homework intervention—with no professional development on the part of the teacher—can positively impact students’ engagement, achievement, and motivation in physics.

## Implications

The development of the WebAssign intervention is one that could be used by other teachers to try to better engage students, particularly female students, in physics. Given the research design, it was not possible to tease out the effects of choice and the combination of choices of biology, sports, and traditional options. A different design that separates out these variables is needed to know specifically what was most motivating for the students. The use of video analysis questions is an area for future research that wasn’t fully explored in this study and how students engage with the problems and its potential needs further investigation. It certainly seems, given the results, that some experience navigating with data gathering in video problems would enhance student achievement on these problems, and the potential for making physics more interesting with video questions ought to be explored. We also want to understand more about why the females had greater gains than the males, despite high interest by the males. We wonder how much could be achieved if this sort of homework set could be used throughout the year, and biology examples were used in class, in addition to traditional examples. We believe that our findings give some promising insights into closing the gender gap in the achievement high school physics students.

## Data Availability

The datasets generated for this study are available on request to the corresponding author.

## Ethics Statement

The Internal Review Board (IRB) at the university (NCSU) approved the proposed study on February 10th, 2016, Protocol #6552. Once IRB approved this study, a letter was sent out to each school’s administration and cooperating teacher. Permission for the research to be conducted in the school was sought from principals and any administration at the district level that was needed. Students were given a consent form and a letter to take home to their parents explaining the research and the student’s role in the research inviting them to participate. The letter consisted of the project description and an invitation for both students and parents. The signed written consent form was returned by the student to the teacher. The cooperating teacher returned all the signed forms to the first author. Teachers were also asked to complete a signed consent forms for their part in the study and these were returned to the lead researcher with the student forms. Teachers were provided with self-addressed stamped envelopes to return all the materials. Students were asked to complete a pre and post survey which could have made them feel uncomfortable, but the results of the surveys were collected online and no one else could see the students’ responses except the researchers. Academic risk was minimized and potential stress from the automatic grading done by WebAssign was mitigated by giving students five chances to get each question correct. Students were also given the option of getting an extension on the assignment if they wanted more time. These measures were taken to avoid any potential risk or stress due to the academic nature of the assignment. The scores students received on these assignments weren’t part of their class grade.

## Author Contributions

Both authors contributed to this research and are accountable for the content of this work.

### Conflict of Interest Statement

The authors declare that the research was conducted in the absence of any commercial or financial relationships that could be construed as a potential conflict of interest.

## Footnotes

^1^www.webassign.net
